# Adaptation to an Amoeba Host Leads to Pseudomonas aeruginosa Isolates with Attenuated Virulence

**DOI:** 10.1128/aem.02322-21

**Published:** 2022-03-08

**Authors:** Wai Leong, Wee Han Poh, Jonathan Williams, Carla Lutz, M. Mozammel Hoque, Yan Hong Poh, Benny Yeo Ken Yee, Cliff Chua, Michael Givskov, Martina Sanderson-Smith, Scott A. Rice, Diane McDougald

**Affiliations:** a Singapore Centre for Environmental Life Science Engineering, Nanyang Technological University, Singapore; b Illawarra Health and Medical Research Institute, Wollongong, Australia; c School of Chemistry and Molecular Biosciences, Molecular Horizons, University of Wollongong, Wollongong, Australia; d The iThree Institute, University of Technology Sydney, Sydney, Australia; e School of Biological Sciences, Nanyang Technological University, Singapore; f Costerton Biofilm Centre, Department of Immunology and Microbiology, University of Copenhagen, Copenhagen, Denmark; Novo Nordisk Foundation Center for Biosustainability

**Keywords:** *Pseudomonas*, biofilm, coadaptation, evolution, predation, protozoa, virulence factors

## Abstract

The opportunistic pathogen Pseudomonas aeruginosa is ubiquitous in the environment, and in humans, it is capable of causing acute or chronic infections. In the natural environment, predation by bacterivorous protozoa represents a primary threat to bacteria. Here, we determined the impact of long-term exposure of P. aeruginosa to predation pressure. P. aeruginosa persisted when coincubated with the bacterivorous Acanthamoeba castellanii for extended periods and produced genetic and phenotypic variants. Sequencing of late-stage amoeba-adapted P. aeruginosa isolates demonstrated single nucleotide polymorphisms within genes that encode known virulence factors, and this correlated with a reduction in expression of virulence traits. Virulence for the nematode Caenorhabditis elegans was attenuated in late-stage amoeba-adapted P. aeruginosa compared to early-stage amoeba-adapted and nonadapted counterparts. Further, late-stage amoeba-adapted P. aeruginosa showed increased competitive fitness and enhanced survival in amoebae as well as in macrophage and neutrophils. Interestingly, our findings indicate that the selection imposed by amoebae resulted in P. aeruginosa isolates with reduced virulence and enhanced fitness, similar to those recovered from chronic cystic fibrosis infections. Thus, predation by protozoa and long-term colonization of the human host may represent similar environments that select for similar losses of gene function.

**IMPORTANCE** Pseudomonas aeruginosa is an opportunistic pathogen that causes both acute infections in plants and animals, including humans, and chronic infections in immunocompromised and cystic fibrosis patients. This bacterium is commonly found in soils and water, where bacteria are constantly under threat of being consumed by bacterial predators, e.g., protozoa. To escape being killed, bacteria have evolved a suite of mechanisms that protect them from being consumed or digested. Here, we examined the effect of long-term predation on the genotypes and phenotypes expressed by P. aeruginosa. We show that long-term coincubation with protozoa gave rise to mutations that resulted in P. aeruginosa becoming less pathogenic. This is particularly interesting as similar mutations arise in bacteria associated with chronic infections. Importantly, the genetic and phenotypic traits possessed by late-stage amoeba-adapted P. aeruginosa are similar to those observed in isolates obtained from chronic cystic fibrosis infections. This notable overlap in adaptation to different host types suggests similar selection pressures among host cell types as well as similar adaptation strategies.

## INTRODUCTION

Many virulence traits of microorganisms are regulated in response to the environment in order to obtain resources, defend against predation by heterotrophic protists, establish a replication niche, or invade a host. The evolution of virulence is a long-standing subject of investigation with important implications for human health. Most opportunistic pathogens are not transmitted person to person but rather transit through the environment between hosts, and therefore, it is unlikely that virulence traits evolved in the host (1–3); rather, it is more likely that these traits evolved in the environment.

Predation by protists, or protozoa, is a major mortality factor for bacteria in the environment (4). Virulence traits that cause human disease are hypothesized to have evolved in response to, and are maintained by, predation pressure, which supports the “coincidental evolution” hypothesis. This hypothesis states that virulence is a coincidental consequence of adaptation to other ecological niches (5–7). Coincidental evolution is supported by examples of factors that play roles in both grazing resistance and virulence toward mammalian cells (7–10), including traits such as cell surface alterations, increased swimming speed, toxin secretion, and biofilm formation (5, 7). Conversely, virulence traits may be attenuated or lost when organisms adapt to form a more commensal relationship with a host (11–14). Microorganisms may also develop specific virulence traits against a specific host becoming a specialist pathogen. Although there are many hypotheses for how virulence traits evolve, there have been few experimental evolution studies on the adaptation of specific virulence traits to different host types and environments (15–18). Such studies are particularly important for understanding how opportunistic pathogens evolve (19).

Pseudomonas aeruginosa is a versatile opportunistic pathogen found in a wide variety of natural habitats. P. aeruginosa has a large (approximately 6.3-Mb) genome containing many genes for metabolism and antibiotic resistance (20), coupled with a complex regulatory network; thus, this organism is able to survive in a variety of niches. It is an important human pathogen, responsible for both acute nosocomial infections (21) and chronic infections, e.g., in the lungs of cystic fibrosis (CF) patients (22). In the CF lung, it has been shown to adapt to a more commensal lifestyle by altering the expression of acute virulence traits such as motility, quorum sensing, and toxin production (23).

While there are many studies addressing the evolution of P. aeruginosa in the CF lung (23–25), less is known about the impact of environmental factors such as protozoan predation on the evolution of virulence. To address this lack of knowledge, here we investigated the adaptation of P. aeruginosa during long-term coincubation with the amoeba Acanthamoeba castellanii. P. aeruginosa was coevolved with A. castellanii for 42 days, and the impact of coevolution was assessed for a range of phenotypes, including virulence in a Caenorhabditis elegans infection model. Adapted populations as well as selected isolates were also sequenced to investigate the range of mutations that occurred during coincubation.

## RESULTS

Here, we examined the effects of long-term coadaptation of P. aeruginosa to the amoeba A. castellanii. Triplicate populations of P. aeruginosa were coadapted with and without A. castellanii for 42 days in M9 minimal medium. Nonadapted control isolates were incubated in M9 medium, while intracellular adapted isolates were collected from amoebae every 3 days. Phenotypic changes of adapted and nonadapted isolates were determined using various assays, as indicated below. To identify the underlying genetic changes associated with these phenotypes, day 3 and 42 populations and single isolates were sequenced.

### Effect of long-term adaptation on virulence factor production.

**FIG 1:**
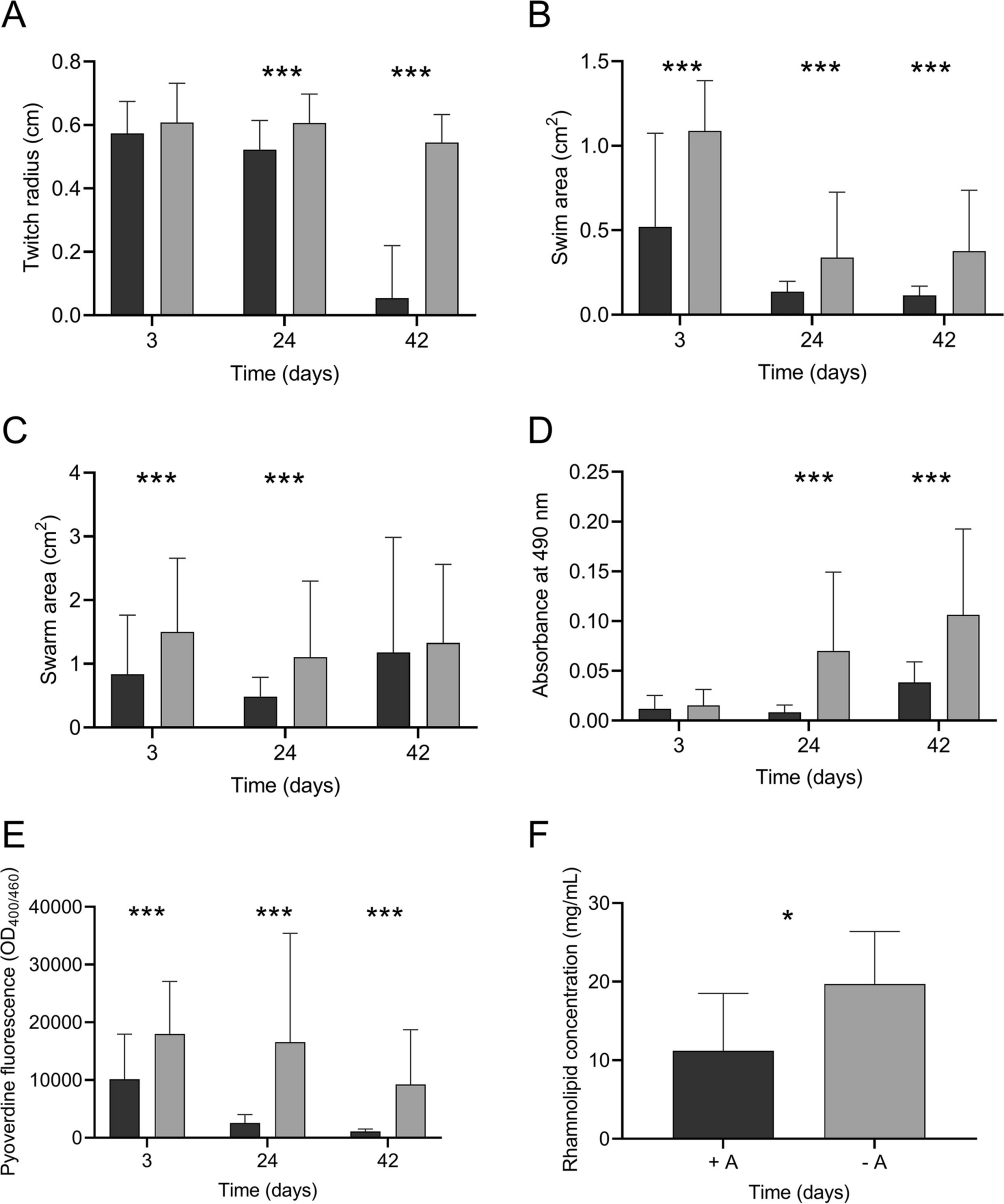
Effect of coincubation on virulence factor production Twitching (A), swimming (B), and swarming (C) motility, biofilm biomass (D), and pyoverdine production (E) of amoeba-adapted (black) and nonadapted (gray) P. aeruginosa isolates from days 3, 24, and 42. Rhamnolipid production of day 42 adapted and nonadapted isolates (F). Data are means and SEM. Statistical analyses for panels A, B, C, D, and E were performed using two-way ANOVA and Tukey’s multiple-comparison test. For panel F, statistical analyses were performed using an unpaired *t* test. *, *P* < 0.05; ***, *P* < 0.001.

To identify alterations in phenotypes expressed by amoeba-adapted and nonadapted isolates, motility, biofilm formation, and pyoverdine and rhamnolipid production were assessed. Nine randomly selected individual isolates of P. aeruginosa, each from adapted and nonadapted populations on days 3, 24, and 42, were assessed. The long-term coincubation of P. aeruginosa with amoebae resulted in a reduction in twitching motility (*F*_2, 534_ = 295.1, *P < *0.001) (Fig. 1A). Amoeba-adapted and nonadapted P. aeruginosa isolates from day 3 did not differ significantly (*P = *0.53); however, on days 24 and 42, twitching motility was significantly reduced compared to that in the nonadapted isolates (*P < *0.001). The mean twitching motility of amoeba-adapted isolates was 10-fold less than isolates that were incubated in the absence of A. castellanii (*P < *0.001).

Coincubation with A. castellanii also resulted in a decrease in swimming motility (*F*_2, 534_ = 15.6, *P < *0.001), where amoeba-adapted isolates from day 3 showed a swim area half that of nonadapted isolates (*P < *0.001) (Fig. 1B). This pattern of reduced swimming motility was also observed for amoeba-adapted isolates from days 24 and 42 (*P < *0.001 and *P < *0.001, respectively). P. aeruginosa isolates also demonstrated a reduction in swarming motility as a result of coincubation with amoebae, which varied over time in a nonlinear fashion (*F*_2, 534_ = 7.597, *P < *0.001) (Fig. 1C). Swarming was significantly reduced in amoeba-adapted isolates on days 3 and 24 (*F*_1, 534_ = 21.73, *P < *0.001). *Post hoc* analysis showed that after 3 days of coincubation, the swarming distance of nonadapted isolates of P. aeruginosa was twice that of amoeba-adapted isolates (*P < *0.001). The swarming distance exhibited by isolates derived from amoeba-adapted and nonadapted isolates on day 24 was further reduced, with a significant reduction in swarming of the amoeba-adapted isolates compared to nonadapted isolates (*P < *0.001). On day 42 there was no significant difference in the average swarming motility between isolates (*P = *0.189).

Coincubation of P. aeruginosa with A. castellanii had a significant effect on P. aeruginosa biofilm formation (*F*_2, 354_ = 15.7, *P < *0.001) (Fig. 1D). *Post hoc* analysis revealed no differences between treatments for day 3 isolates (*P = *0.998). However, amoeba-adapted isolates from day 24 formed 10-fold less biofilm than the nonadapted isolates (*P < *0.001). Although the average biomass of biofilms formed by the amoeba-adapted isolates increased after day 42, biofilm biomass remained 2-fold lower than that of the nonadapted isolates (*F*_1, 354_ = 29.6, *P < *0.001).

A. castellanii*-*adapted P. aeruginosa isolates showed reduced production of pyoverdine compared to nonadapted isolates (*F*_1, 174_ = 45.74, *P < *0.001) (Fig. 1E). Although pyoverdine production was reduced in both amoeba-adapted and nonadapted isolates (*F*_2, 174_ = 12.08, *P < *0.001), the concentration of pyoverdine in supernatants from amoeba-adapted isolates from day 3 isolates was reduced 2-fold compared to that of nonadapted isolates (*P < *0.001) and was further reduced on days 24 and 42 (*P < *0.001). Rhamnolipid production of the amoeba-adapted and nonadapted isolates varied on day 42 (Fig. 1F). Amoeba-adapted isolates produced less rhamnolipid overall than the nonadapted isolates (*t*_16_ = 2.571, *P = *0.0205).

### Amoeba-adapted P. aeruginosa isolates showed reduced virulence in C. elegans.

**FIG 2:**
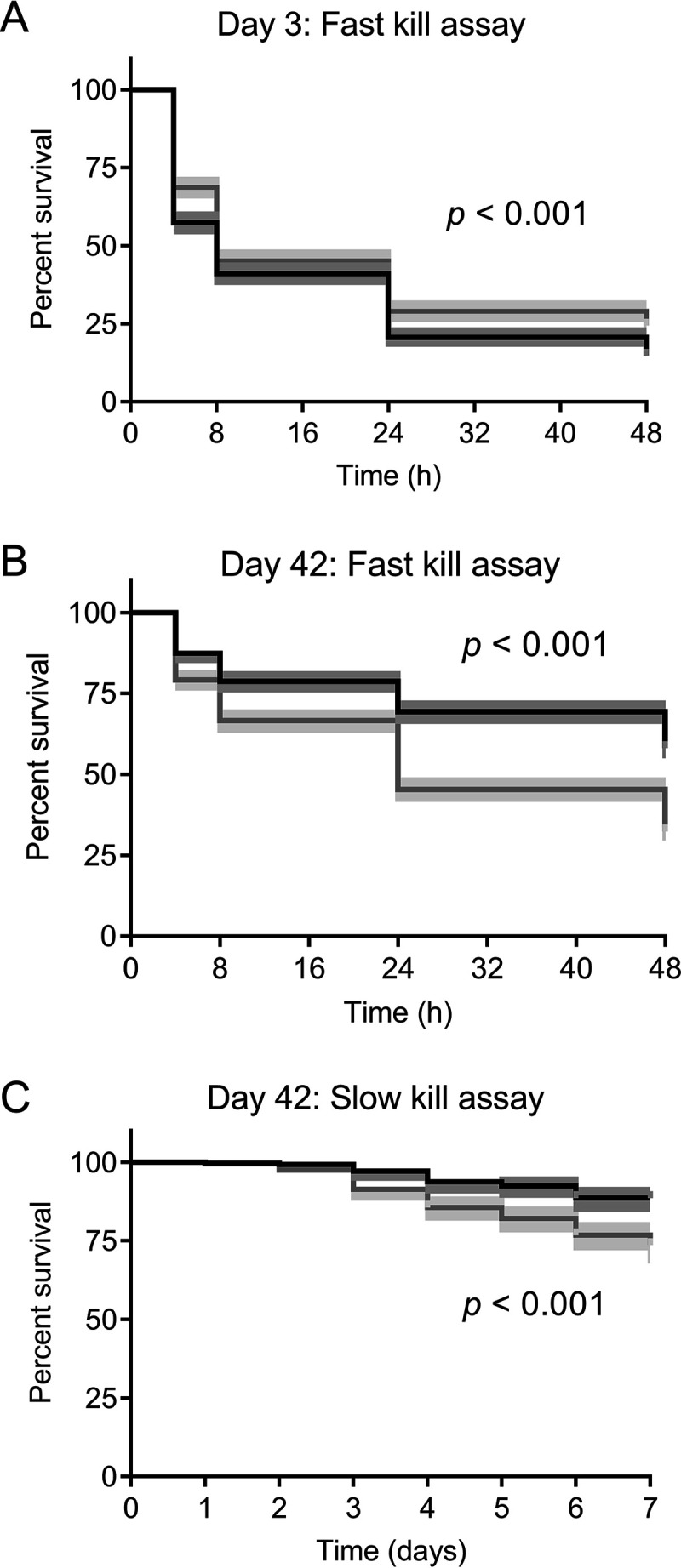
Effect of coincubation of P. aeruginosa with A. castellanii on virulence to C. elegans. C. elegans survival curves after exposure to P. aeruginosa isolates derived from amoeba-adapted (black) or nonadapted (gray) isolates taken from days 3 (A) and 42 (B) for 0, 4, 8, 24 and 48 h in a fast-kill assay. (C) Percent survival of C. elegans exposed to day 42 amoeba-adapted and nonadapted isolates of P. aeruginosa over 7 days in a slow-kill assay. Statistical significance between adapted and nonadapted isolates was determined using log-rank tests. The shaded area represents the 95% confidence interval (CI).

Most of the phenotypes discussed above play a role in the pathogenesis of P. aeruginosa. Since the amoeba-adapted isolates showed marked reduction in the expression of multiple virulence phenotypes, we tested them for virulence in C. elegans fast- and slow-kill assays (Fig. 2A to C). The day 3 amoeba-adapted isolates were significantly more toxic to nematodes than nonadapted isolates, although nematodes exposed to isolates from both populations had a median survival of 8 h (*P < *0.001) (Fig. 2A). C. elegans organisms feeding on day 42 isolates survived longer than those feeding on isolates from day 3. Furthermore, amoeba-adapted isolates from day 42 were significantly less toxic to C. elegans than their nonadapted counterparts in both fast-kill (Fig. 2B) (*P < *0.001) and slow-kill (Fig. 2C) assays (*P < *0.001).

### Genotypic changes in A. castellanii-adapted and nonadapted P. aeruginosa.

**FIG 3:**
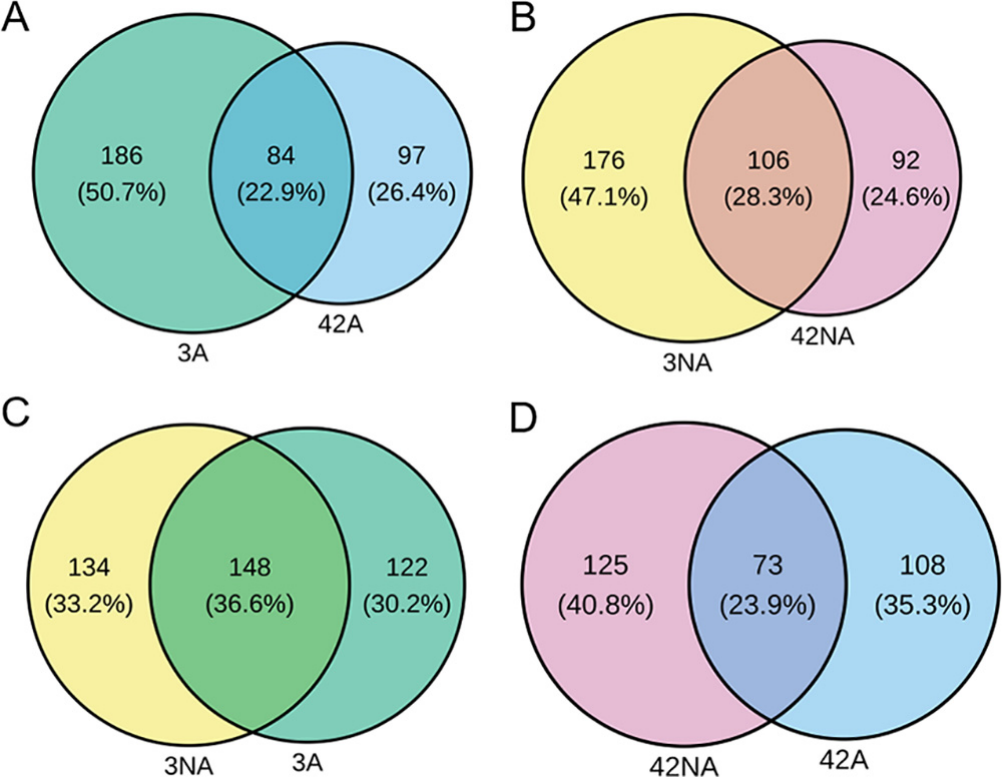
Unique and shared mutated genes in coding regions of different populations. Venn diagrams of the numbers and percentages of unique and shared genes in day 3 versus 42 adapted (3A and 42A) (A), day 3 versus 42 nonadapted (3NA and 42NA) (B), day 3 adapted versus nonadapted (C), and day 42 adapted versus nonadapted (D) populations.

To identify the underlying genetic basis for the attenuated virulence and reduced production of virulence factors, three replicate populations (L1, L2, and L3) of amoeba-adapted and nonadapted P. aeruginosa from days 3 and 42 were sequenced (see Data Sets S1 and S2 in the supplemental material). In addition to whole-population sequencing, 11 amoeba-adapted single isolates and 3 nonadapted single isolates of P. aeruginosa from day 42 were sequenced and analyzed (see Data Sets S3 and S4). The numbers of single nucleotide polymorphisms (SNPs), both synonymous (sSNPs) and nonsynonymous (nsSNPs), as well as insertions and deletions (indels) and intergenic mutations occurring in amoeba-adapted and nonadapted populations and isolates were determined (Fig. S1A and B). More mutations were observed in both day 3 amoeba-adapted (*n* = 511) and nonadapted (*n* = 601) populations than day 42 amoeba-adapted (*n* = 327) and nonadapted (*n* = 383) populations. This strongly suggests adaptive evolution of P. aeruginosa, as random mutations arise during early stages of adaptation and only beneficial mutations become fixed in the later populations due to selection pressure. The day 3 and 42 adapted populations had 356 and 227 mutations in coding regions (sSNPs, nsSNPs, and indels) corresponding to 270 and 181 genes, respectively. In contrast, day 3 and 42 nonadapted populations had 424 and 271 mutations in coding regions (sSNPs, nsSNPs, and indels) corresponding to 282 and 198 genes, respectively. Day 3 and 42 adapted and nonadapted populations shared 22.9% (*n* = 84) and 28.3% (*n* = 106) mutated genes between them, respectively (Fisher’s exact test, *P = *0.09) (Fig. 3A and B). There were more shared genes (*n* = 148; 36.6%) in day 3 adapted and nonadapted populations compared to day 42 adapted and nonadapted populations (*n* = 73, 23.9%) (Fisher’s exact test, *P < *0.001) (Fig. 3C and D).

**FIG 4:**
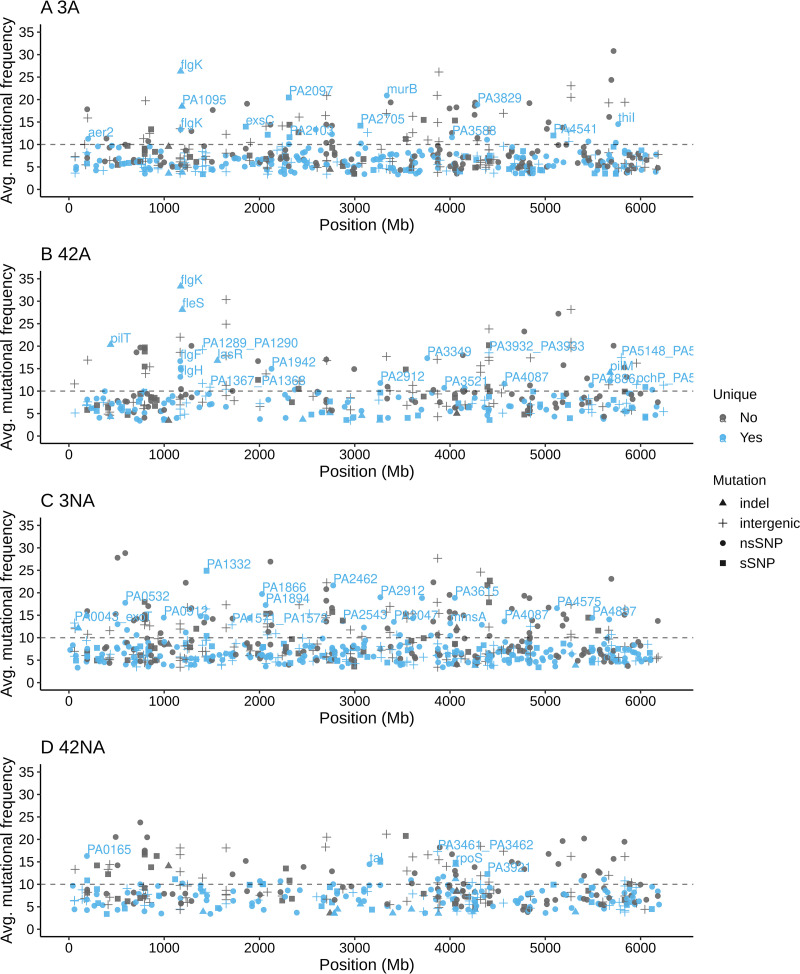
Mutations in amoeba-adapted and nonadapted P. aeruginosa on days 3 and 42. The average mutational frequencies of three replicate populations (L1, L2, and L3) of day 3 adapted (A), day 42 adapted (B), day 3 nonadapted (C), and day 42 nonadapted (D) populations are plotted along the P. aeruginosa genome. Different symbols represent different types of mutation. The unique mutations (those detected in either adapted or nonadapted populations) are blue, while gray denotes mutations found in all groups.

Many mutations in known virulence-related genes were observed in both day 3 and day 42 adapted populations but not in the nonadapted populations (Table 1 and Fig. 4). A large number of motility-related genes were mutated in adapted populations, most notably in flagellar (*flgK*, *fleS*, *flgF*, and *flgH*) and type IV pilus genes (*pilI*, *pilM*, *pilN*, *pilR*, and *pilT*). Genes involved in pyoverdine synthesis (*pvdN*) as well as the quorum sensing regulator *lasR* were mutated only in day 42 adapted populations. Only 14 mutations that occurred in day 3 adapted lineages (L1, L2, and L3) were maintained in the day 42 population (Fig. S2). Among those persistent mutations, the mutational frequency increased from day 3 to day 42 in adapted populations for *flgK* (78.9% to 100% in L3 and 40% to 45.5% in L2), *fleS* (11.3% to 84.4% in L2), and PA2069 (23% to 27.4% in L1). In contrast, 21 different mutations were maintained from day 3 to 42 in nonadapted populations (Fig. S3).

**TABLE 1:** nsSNPs and indels found in known virulence factor-encoding genes that occurred solely in day 3 and 42 adapted populations

Gene/locus tag	Function	Mutation^*a*^	Result on day^*b*^:	
			3	42
Motility/adherence				
*pilT*/PA0395	Twitching motility protein PilT	Δ1 bp (100)	−	+
		Δ12 bp (570–581)		
*pilI*/PA0410	Twitching motility protein PilI	V108G (GTG→GGG)	+	+
		V109G (GTG→GGG)		
		V123G (GTG→GGG)		
*flgF*/PA1081	Flagellar basal body rod protein FlgF	E188* (GAG→TAG)	−	+
*flgH*/PA1083	Flagellar basal body L-ring protein	G163C (GGC→TGC)	−	+
flgK/PA1086	Flagellar hook-associated protein FlgK	Δ1 bp (797)	+	+
		Y394* (TAC→TAG)		
*fliS*/PA1095	B-type flagellar protein FliS	Δ2 bp (273–274)	+	−
*fleS*/PA1098	Two-component sensor	T154P (ACC→CCC)	+	+
*pilR*/PA4547	Two-component response regulator PilR	E253D (GAA→GAC)	−	+
*pilN*/PA5043	Type 4 fimbrial biogenesis protein PilN	E148* (GAG→TAG)	−	+
*pilM*/PA5044	Type 4 fimbrial biogenesis protein PilM	Δ2 bp (577–578)	−	+

Quorum sensing				
*lasR*/PA1430	Transcriptional regulator LasR	Δ38 bp (480–517)	−	+

Iron uptake				
*pvdN*/PA2394	Pyoverdine biosynthesis protein PvdN	L404P (CTG→CCG)	−	+
*pvdL*/PA2424	Peptide synthase	A4026P (GCC→CCC)	+	−
*fptA*/PA4221	Fe(III)-pyochelin outer membrane receptor	T239P (ACC→CCC)	+	−

a Δ, deletion mutation. nsSNPs are indicated with the position of the affected amino acid, and respective base changes are underlined. *, presence of translation termination (stop) codon.

b + and −, presence and absence of the respective mutations. The genes are classified according to the virulence factor database (http://www.mgc.ac.cn/VFs/main.htm).

Mutational analysis of day 42 adapted (*n* = 11) and nonadapted (*n* = 3) single isolates of P. aeruginosa revealed a total of 107 and 60 mutations affecting 51 and 25 genes, respectively (Fig. S1B). Twenty-four of 51 genes (47%) mutated in day 42 adapted isolates encoded virulence factors according to the virulence factor database (http://www.mgc.ac.cn/cgi-bin/VFs/genus.cgi?Genus=*Pseudomonas*). The mutated genes encoding virulence factors were classified into different functions, including motility, quorum sensing, metabolism, and protease production (Table 2). Genes related to motility and adherence, flagella (*flgF*, *flgH*, *flgK*, and *fliS*) and type IV pili (*pilJ*, *pilM*, *pilN*, and *pilT*), were mostly mutated at 100% frequency. In addition, *lasR* was also mutated at 100% frequency. The genes involved in motility and quorum sensing (*pilJ*, *pilM*, *pilT*, *flgK*, *fliS*, *fleS*, and *lasR*) harbor deletion mutations ranging from 1 to 399 bp.

**TABLE 2:** nsSNP and indel found in genes with known function that occurred solely in day 42 amoeba-adapted isolates

Gene/locus tag	Function	Mutation^*a*^
Motility/adherence		
*pilT*/PA0395	Twitching motility protein PilT	Δ45 bp (78–122) #
		Δ1 bp (100) #
		Δ12 bp (570–581) #
*pilJ*/PA0411	Twitching motility protein PilJ	Δ399 bp (1153–1551) #
*flgF*/PA1081	Flagellar basal body rod protein FlgF	E188* (GAG→TAG) #
*flgH*/PA1083	Flagellar basal body L-ring protein	G163C (GGC→TGC) #
*flgK*/PA1086	Flagellar hook-associated protein FlgK	Δ1 bp (797/2,052 nt) #
		Y394* (TAC→TAG) #
*fliS*/PA1095	B-type flagellar protein FliS	Δ8 bp (159–166) #
*fleS*/PA1098	Two-component sensor	Δ17 bp (675–691) #
*pctA*/PA4309	Chemotactic transducer PctA	N593Y (AAC→TAC)
*pilN*/PA5043	Type 4 fimbrial biogenesis protein PilN	E148* (GAG→TAG) #
*pilM*/PA5044	Type 4 fimbrial biogenesis protein PilM	Δ1 bp (584) #

Quorum sensing		
*lasR*/PA1430	Transcriptional regulator LasR	Δ38 bp (480–517) #

Metabolism/energy production		
*glcB*/PA0482	Malate synthase G	H467Y (CAC→TAC)
PA1617	AMP-binding protein	L168P (CTC→CCC)
		L171V (CTG→GTG)
		L171Q (CTG→CAG)
*nirB*/PA1781	Assimilatory nitrite reductase	Q84* (CAG→TAG)
PA2119	Alcohol dehydrogenase	N306Y (AAT→TAT)
		T (912)
*spdH*/PA3713	Spermidine dehydrogenase SpdH	A464T (GCG→ACG)
PA4292	Phosphate transporter	V42D (GTC→GAC)
*arcA*/PA5171	Arginine deiminase	K197T (AAG→ACG)

Defense mechanism		
PA2735	Restriction-modification system protein	A546T (GCC→ACC)

Protease		
PA1327	Protease	V484I (GTC→ATC)

Other		
PA1181	Sensor protein	R544C (CGC→TGC)
*grx*/PA5129	Glutaredoxin	M1T (ATG→ACG)
PA3047	d-Alanyl-d-alanine carboxypeptidase	F96I (TTC→ATC)
PA5210	Secretion pathway ATPase	T358A (ACG→GCG)
		T358K (ACG→AAG)
		L359I (CTC→ATC)

a Δ, deletion. nsSNPs are indicated with the positions of the affected amino acids, and respective base changes are underlined. *, presence of a translation termination (stop) codon; #, mutational frequencies at 100%. The genes are classified according to the virulence factor database (http://www.mgc.ac.cn/VFs/main.htm).

### Adapted isolates showed increase competitive fitness and survival in amoebae and macrophages.

**FIG 5:**
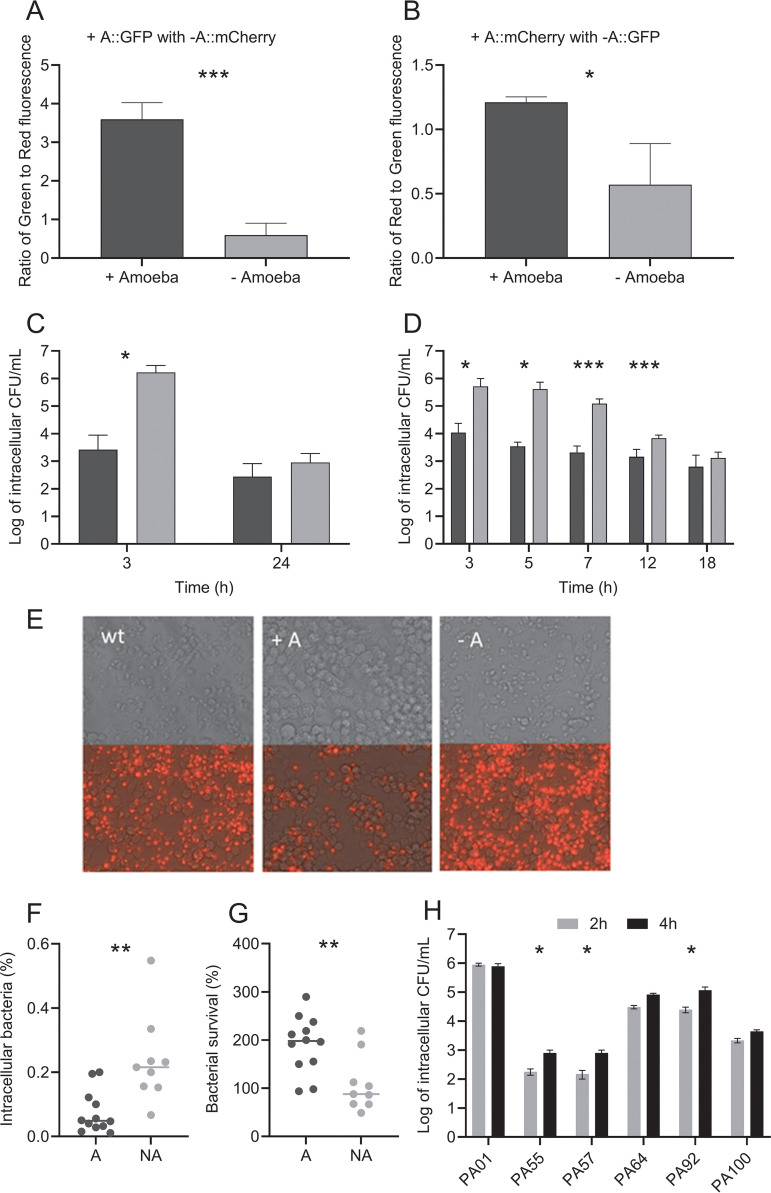
Competition and intracellular survival assays of amoeba-adapted and nonadapted isolates. The fluorescence ratios of day 42 amoeba-adapted (+A::GFP) mixed with nonadapted (−A::mCherry) P. aeruginosa (A) and amoeba-adapted (+A::mCherry) with nonadapted (−A::GFP) P. aeruginosa (B) after 48 h of incubation with (black bars) and without (gray bars) A. castellanii. Intracellular survival of day 42 amoeba-adapted (black) and nonadapted (gray) isolates is shown as CFU mL^−1^ over time in a modified gentamicin protection assay (log scale; *n* = 3) conducted with amoebae (C) and macrophages (D). Propidium iodide staining of RAW 264.7 macrophages 24 h after infection with wild-type (wt), day 42 amoeba-adapted (A+), and nonadapted (−A) P. aeruginosa (E). Images are shown with and without the fluorescence to illustrate changes in cell morphology. Survival of day 42 amoeba-adapted (A) and nonadapted (NA) strains following incubation with human neutrophils where bacterial uptake (F) and survival (G) were determined. Intracellular numbers of CF isolates at 2 and 4 h of infection with amoebae were determined (H). The 4-h/2-h ratios were used to determine the significance of intracellular survival compared to that of PAO1 using the Kruskal-Wallis test. Data are means and SEM. Groups were analyzed by Student's *t* test. *, *P* < 0.05; **, *P* < 0.01; ***, *P* < 0.001.

To investigate whether adaptation with amoeba confers a fitness advantage to P. aeruginosa, we mixed fluorescence-tagged amoeba-adapted and nonadapted isolates and grew them together with amoebae. After 48 h of coincubation, the proportion of amoeba-adapted cells was always higher (amoeba-adapted cells tagged with green fluorescent protein [amoeba-adapted::GFP] [*F*_1, 4_ = 95.27, *P = *0.000617] and amoeba-adapted cells tagged with mCherry [amoeba-adapted::mCherry] [*F*_1, 4_ = 11.85, *P = *0.0262]) when competed with the reciprocally tagged nonadapted strain than with no-amoeba controls (Fig. 5A and B). In addition, the intracellular survival of day 42 adapted and nonadapted isolates was determined using a modified gentamicin protection assay. Numbers of intracellular CFU 3 h after infection of nonadapted isolates in amoeba were higher than those of amoeba-adapted CFU; however, after 24 h, the numbers of surviving intracellular nonadapted cells had decreased and were comparable to the amoeba-adapted numbers (adaptation × time *F*_1, 32_ = 14, *P < *0.001) (Fig. 5C).

### Adapted isolates exhibit reduced uptake by and enhanced survival within different phagocytic cells.

In order to determine if the intracellular survival of amoeba-adapted isolates extends to other phagocytic cell types, we compared the ability of adapted and nonadapted P. aeruginosa to survive in the presence of macrophage and human neutrophils. When the assay was conducted with macrophages, survival trends were similar to that observed with amoeba (Fig. 5D). There was a significant interaction of amoeba adaptation and incubation time (adaptation × time *F*_4, 64_ = 6.692, *P < *0.001), with a higher initial uptake of day 42 nonadapted isolates than of amoeba-adapted isolates, resulting in higher initial numbers of intracellular CFU, followed by a constant decrease in viable intracellular numbers between 5 and 18 h postinfection. The day 42 amoeba-adapted isolates were initially taken up by macrophages in lower numbers, and the number of viable intracellular CFU did not decrease to the same extent as the nonadapted isolates, resulting in comparable numbers at 18 h postinfection. At 24 h postinfection, macrophages infected with nonadapted P. aeruginosa exhibited morphological changes and appeared similar to those infected with the wild-type strain (Fig. 5E). Propidium iodide staining showed that many of these macrophages were dead. In contrast, macrophages infected with amoeba-adapted P. aeruginosa exhibited a more normal morphology, with fewer cells taking up the propidium iodide stain, suggesting that amoeba-adapted isolates were less toxic to the macrophages than the nonadapted isolates. Assays with human neutrophils showed that, after 2 h infection, intracellular CFU of nonadapted isolates in neutrophils were higher than amoeba-adapted CFU (Fig. 5F). Amoeba-adapted isolates also showed increased survival in the presence of human neutrophils compared to nonadapted isolates (Fig. 5G).

### Chronic cystic fibrosis isolates also exhibit enhanced survival and reduced uptake.

As late-stage amoeba-adapted isolates exhibit several phenotypes similar to those of chronic CF isolates, we wanted to know whether CF isolates also behave similarly in terms of intracellular survival and uptake by amoebae. We tested the uptake and intracellular survival of five CF isolates (PA55, PA57, PA64, PA92, and PA100) in amoebae and compared them with those of PAO1. The results showed that the CF isolates showed reduced uptake and increased intracellular survival, similar to the amoeba-adapted isolates (Fig. 5H). All the CF isolates tested showed reduced uptake by amoebae. The majority of the CF isolates (PA55, PA57, and PA92) showed a significant increase in survival compared to PAO1.

## DISCUSSION

In this study, amoeba-adapted isolates displayed reductions in many virulence phenotypes, including loss of motility and reductions in pyoverdine and rhamnolipid production. These altered virulence phenotypes are very similar to phenotypes of P. aeruginosa isolates from CF lineages, which include gains in mucoidy and antibiotic resistance and loss of secondary metabolites and motility (23). The parallel losses of motility and secondary metabolites in amoeba-adapted isolates in this study are particularly striking and evoke the question of whether the selective forces driving these traits are the same in both systems.

Strong negative selection against traits could occur due to host recognition and the need for evasion by pathogens. In both cases, loss of motility appears to be a strong selection factor. This is because both mammalian immune cells and amoebae can recognize and bind P. aeruginosa flagella through surface receptors (26, 27). Furthermore, loss of motility has also been shown to significantly reduce phagocytic uptake by mammalian immune cells (28). Several findings presented here also support predator avoidance as a selection pressure. Uptake and intracellular survival experiments with different phagocytic cells revealed that nonadapted isolates exhibit chemotaxis and rapidly swim toward and are taken up by amoebae and macrophages (Fig. 5C and D). However, we observed that amoeba-adapted isolates do not attach to the surface of the amoeba. This is supported by experiments showing reduced uptake by amoebae and macrophages (Fig. 5C and D). Loss of flagella and motility is therefore adaptive for the purpose of predator avoidance. Additionally, chemotaxis mutants in PA3348 were detected in the population genomic data. The loss of chemotaxis in amoeba-adapted strains would be consistent with predator avoidance.

Traits may also be lost if the costs of maintaining the traits outweigh the benefits. Loss of pyoverdine in P. aeruginosa in chronic CF infections has been reported (29) and shown to be driven by social selection (30). In these cases, non-pyoverdine-producing cheats benefit by retaining the pyoverdine receptor for uptake of pyoverdine and iron, without needing to produce the metabolically expensive product. Only when extrinsic pyoverdine is completely lost do mutations appear in receptor genes.

In our study, there were many *pvd* mutations; however, we did not observe any mutations in the receptor genes. This suggests that social selection may also occur. It is also possible that other avenues of iron uptake are preferentially utilized (31), as iron uptake occurs via *hemO* in late CF strains (32). Selection against pyoverdine production may be a balance between the effect of the energy cost of pyoverdine synthesis, iron uptake, and oxidative stress as previously described. Thus, the cost of maintaining the traits may outweigh the benefits, resulting in the selection against these genes.

In addition to the phenotypes described above, some of the most common mutations in various models of infection and coevolution studies occur in quorum sensing genes. For example, mutations in *lasR* are common in CF isolates (33). Such mutations are also found in coevolution studies with C. elegans, where mutations in *lasR* and *rhlR* quorum sensing genes were found to occur early in the adaptation process. The regulatory genes *lasR* and *rhlR* control the expression of many virulence genes. In turn, *las* and *rhl* mutants have been shown to be less virulent in models of wound infection (34). In contrast, no correlation between *lasR* and loss of virulence was found in a later study investigating virulence using a Dictyostelium discoideum host model and 13 CF isolates harboring mutations in *lasR* (35). The study does not formally rule out an influence of *lasR* inactivation on virulence, although they suggested that *lasR* mutations may represent only a minor factor in the evolution of bacterial virulence. Mutations in *lasR* affect many downstream genes and quorum sensing systems, and it has been proposed that such pleiotropic adaptive mutations in global regulatory genes are more likely to occur than multiple mutations in individual virulence traits. This may account for the prevalence of *lasR* mutations in chronic CF mutations. In this study, mutations in *lasR* occurred in only one of the three amoeba-adapted replicates, suggesting that a stronger selection may drive the loss of individual virulence traits in the other amoeba-adapted populations.

Experiments with both amoebae and phagocytic immune cells in this study also suggest that fitness gains from adapting to amoebae could be similarly conferred to interactions with macrophages. In amoebae, macrophages, and neutrophils, amoeba-adapted P. aeruginosa isolates display reduced uptake and virulence. This demonstrates the overlaps in traits used by P. aeruginosa to interact with these hosts. This work also highlights potential overlaps in host-pathogen interaction processes, even between cells as divergent as mammalian macrophages and single-celled amoebae. The A. castellanii genome contains homologues of the gamma interferon-inducible lysosomal thiol reductase (GILT), interferon inducible GTPase, and the NRAMP homologue, all of which play a role in antimicrobial defense in mammalian cells (36). However, more research is needed to determine whether these defenses play a role in P. aeruginosa infection and whether P. aeruginosa possesses mechanisms to evade such defenses in order to survive intracellularly within both phagocytic cell types.

Moreover, while CF macrophages are known to have impaired phagocytosis and bacterial killing, this has been shown to be mainly due to the CF lung environment, inflammation, expression of dysfunctional cystic fibrosis transmembrane conductance regulator (CFTR) in macrophages, and other defects associated with CFTR dysfunction in macrophages and other immune cells (37, 38). In burn wound patients, type III secretion system (T3SS) effectors such as ExoS and ExoT were also implicated in defective macrophage phagocytosis by disrupting host cell actin skeleton (39). In these cases, the loss of flagella, pili, and motility, as observed in our study, may not be the primary driver of reduced uptake of P. aeruginosa. Together, these observations suggest that reduced uptake of P. aeruginosa by predators or immune cells is important for long-term survival and adaptation in amoebae and may provide survival advantages in macrophages. However, the selection for this phenotype likely occurs through various mechanisms.

The loss of function in virulence genes in amoeba-adapted isolates likely leads to a subsequent loss in virulence phenotypes. Similar decreases in acute virulence phenotypes also occur in P. aeruginosa strains isolated from chronically infected CF patients (23, 40, 41). P. aeruginosa in experimental evolution experiments with C. elegans also evolved an attenuated virulence phenotype after serial passages (14). This is in contrast to other pathogens, where virulence has been demonstrated to increase after serial passages (42), once again suggesting that P. aeruginosa may adapt toward a more commensal and chronic lifestyle in coevolution processes.

In this study, it was demonstrated that adaptation to a more commensal lifestyle may also confer benefits in an infectious context for a generalist pathogen, as it is clear that although amoeba-adapted cells are less virulent, they are still capable of invading and colonizing C. elegans. Similarly, adapted CF strains have been shown to be as capable as environmental strains of infecting a new host in a mouse model (43). Although amoebae may be thought of as training grounds for the formation of virulence traits, they may also be grounds for the selection for a more “chronic” state of coexistence. The data presented here support the coincidental evolution hypothesis of virulence, where adaptation to commensal habitats may coincidentally modulate virulence factors. Thus, it is adaptation to environmental niches that drives the evolution of virulence phenotypes and not interaction with the human host.

## MATERIALS AND METHODS

### Organisms and growth conditions.

P. aeruginosa strain DK1, used for this study, was initially obtained from a Danish CF patient (P30M0) (24). Unless otherwise stated, P. aeruginosa DK1 and population-derived isolates were grown in 10 mL lysogeny broth (LB10; BD Biosciences, USA) at 37°C with shaking at 200 rpm. A. castellanii was obtained from the American Type Culture Collection (ATCC 30234) and was routinely maintained axenically in peptone-yeast-glucose (PYG) medium (20 g protease peptone, 5 g yeast extract, and 50 mL 2 M glucose L^−1^) at room temperature. Prior to use in experiments, A. castellanii was passaged and washed twice with 1× phosphate-buffered saline (PBS; Sigma-Aldrich, USA) solution to remove PYG medium. C. elegans N2 Bristol was maintained on nematode growth medium (NGM) (per liter; 2.5 g Bacto-Peptone [BD Biosciences, USA], 3 g NaCl, 7.5 g agar, 1 mL 5 mg mL^−1^ cholesterol, 1 mL 1 M MgSO_4_, 1 M CaCl_2_, and 25 mL 1 M potassium phosphate buffer at pH 6) fed with Escherichia coli OP50. RAW 264.7 macrophages (ATCC TIB-71) were grown in Dulbecco’s modified Eagle medium (DMEM; Thermo Fisher, USA) with 10% fetal bovine serum (FBS) at 37°C with 5% CO_2_. Before use, cells were washed with PBS and treated with trypsin briefly before gentle detachment by scraping. Cells were then centrifuged at 1,000 × *g* for 1 min and resuspended in experimental medium before use.

### P. aeruginosa and A. castellanii coincubation.

Overnight cultures of P. aeruginosa grown in LB10 medium was centrifuged at 4,000 × *g* for 5 min and washed twice with 1× M9 salts solution (Sigma-Aldrich, USA; per liter, 6.78 g Na_2_HPO_4_, 3 g H_2_PO_4_, 1 g NH_4_Cl, 0.5 g NaCl). A. castellanii, at a concentration of 1 × 10^3^ cells mL^−1^, was seeded onto the surfaces of 25-cm^2^ tissue culture flasks with 0.2-μm-vented caps filled with 10 mL 1× complete M9 salts plus 0.01% glucose. To maintain a strong selective pressure from amoebae, 100 μL of A. castellanii and P. aeruginosa was taken from percussed, 3-day established flasks and added to new flasks containing A. castellanii every 3 days. Three biological replicates (L1, L2, and L3) of P. aeruginosa with A. castellanii were maintained and termed amoeba-adapted populations, and the derived isolates were termed amoeba-adapted isolates.

In parallel with the coincubation experiment, three biological replicates of P. aeruginosa were also maintained without A. castellanii and termed nonadapted populations, and derived isolates were termed nonadapted isolates. Briefly, P. aeruginosa was diluted to a cell concentration of 1 × 10^2^ cells mL^−1^ and added to tissue culture flasks containing 10 mL 1× complete M9 plus 0.01% glucose. From these flasks, 100 μL of 3-day-old established P. aeruginosa culture was added to flasks containing fresh medium every 3 days.

### Isolation of intracellular P. aeruginosa.

On days 3, 24, and 42, flasks containing A. castellanii (amoeba adapted) were percussed until the amoebae detached, and 1 mL of the culture medium was filtered through a 3-μm cellulose acetate membrane (Merck, Germany) to retain A. castellanii. A. castellanii organisms were resuspended in 5 mL of 1× M9 salts and pelleted at 4,000 × *g* for 5 min before resuspension in 100 μL 1× M9 salts solution. A. castellanii were lysed by the addition of 100 μL of 1% Triton–X for 1 min; the mixture was then pelleted and washed twice with 900 μL of 1× M9 salts. The cell pellet was resuspended in 1 mL of 70% LB10 plus 30% glycerol and stored at −80°C. The same treatment was applied to the nonadapted P. aeruginosa.

### Phenotypic screening of amoeba-adapted and nonadapted isolates.

To facilitate phenotypic screening, nine single isolates were randomly selected from each amoeba-adapted and nonadapted population on days 3, 24, and 42. Phenotypic changes were determined by motility and biofilm, pyoverdine, and rhamnolipid production assays, as indicated below.

### Biofilm assay.

To determine if adaptation with A. castellanii altered biofilm formation, the biomass of attached cells was quantified by crystal violet staining as previously described (44). Briefly, overnight cultures of each isolate were added to 96-well plates containing 100 μL 1× M9 plus 0.4% glucose. Plates were incubated at room temperature with agitation at 80 rpm for 24 h. To separate the suspended biomass from the attached biomass, each well was washed once with 1× PBS. Cells attached to the plate surface were stained with 200 μL of 0.3% crystal violet and incubated for 20 min, after which unbound crystal violet was removed by washing 3 times with 1× PBS. Crystal violet was liberated from the cells with 300 μL of absolute ethanol. The absorbance of the crystal violet was measured with a spectrophotometer at 590 nm (Infinite M200; Tecan, Switzerland) in triplicate.

### Motility assays.

Twitching, swarming, and swimming motilities were assessed as previously described, using motility agar (20 mM NH_4_Cl, 12 mM Na_2_HPO_4_, 22 mM KH_2_PO_4_, 8.6 mM NaCl, 1 mM MgSO_4_,100 μM CaCl_2_, 2 g L^−1^ dextrose, 5 g L^−1^ Casamino Acids) containing 1, 0.5, or 0.3% (wt vol^−1^) agarose (Bacto; BD Biosciences, USA), respectively (45, 46). Five milliliters of motility agar was added to the wells of 6-well plates and dried under laminar flow for 1 h. Isolates were inoculated into the centers of the wells using 10-μL pipette tips, either to the base of the plate for assessment of twitching motility or the middle of the agar for assessment of swimming and swarming. Twitching and swarming plates were incubated at room temperature for 48 h and swimming plates were incubated for 24 h prior to imaging with a digital camera (Canon EOS 600D digital single-lens reflex [DSLR]) mounted on a tripod, to allow phenotypic characterization of the resulting colonies and comparative endpoint twitching, swarming, and swimming distances. The zone of motility was semiquantitatively determined using ImageJ image analysis software. Motility was assessed in triplicate (*n* = 3).

### Quantification of pyoverdine.

To determine if adaptation with amoebae (3, 24, and 42 days) affects the production of pyoverdine, isolates were grown overnight in 1 mL LB10 medium. Cells were removed by centrifugation at 5,200 × *g* for 5 min, and the absorbance of the supernatant was determined with a spectrophotometer (Infinite M200; Tecan, Switzerland) at an excitation wavelength of 400 nm and an emission wavelength of 460 nm, in triplicate.

### Quantification of rhamnolipids.

The orcinol method (47) was used to quantify the production of rhamnolipid biosurfactant of adapted and nonadapted isolates from the day 42 populations. Briefly, overnight P. aeruginosa LB cultures were diluted to an optical density at 600 nm (OD_600_) of 0.01 in 25 mL of AB minimal medium (48) supplemented with 2 g glucose and 2 g Casamino Acids L^−1^ and grown overnight at 37°C with shaking at 200 rpm. The cell density was determined (OD_600_) before filtration and extraction of crude rhamnolipid from the supernatant twice using diethyl ether (7 mL). The organic layer was collected, combined, and concentrated in a vacuum concentrator (SpeedVac; Thermo Scientific) at 0°C for 1 h followed by 2 h at 25°C, until white solids formed. The solids were resuspended in 500 μL of water, and 50 μL of this solution was mixed with 450 μL of freshly prepared orcinol (0.19% in 53% H_2_SO_4_). Samples were incubated at 80°C for 30 min and allowed to cool at room temperature for 15 min before quantification of absorbance (OD_421_). The absorbance was normalized to cell concentration (OD_600_) for each sample, and a factor of 2.5 was applied to convert values from a rhamnose standard curve to rhamnolipid concentration (49).

### Fluorescent tagging of isolates.

To prepare fluorescently tagged amoeba-adapted and nonadapted P. aeruginosa, two isolates were randomly selected from the day 42 populations and grown overnight at 37°C in LB broth. Electroporation was performed as previously described (50). One milliliter of P. aeruginosa at a cell density of 1 × 10^8^ cells mL^−1^ was pelleted and washed twice with 300 mM sucrose. The expression tag carrying plasmid pUC18-TR6K-mini-Tn7T-Gm-GFP (0.5 μg) (expressing a green fluorescent protein [*gfp*]; emission, 488 nm/excitation, 509 nm) (51) or pUC18T-miniTn7T-Gm-Mcherry (0.5 μg) (expressing a red fluorescent protein [mCherry]; emission, 587 nm/excitation, 610 nm) (52) was mixed with 1 μg pTNS1 helper plasmid and 300 μL resuspended bacteria, in a 2-mm-gap electroporation cuvette. This mixture was electroporated at 1.8 kV, 25 μF, 2,100 Ω, and 2.5 kV cm^−1^ in a Gene Pulser apparatus (Bio-Rad, Hercules, CA, USA). Cell recovery was performed in ice-cold super optimal broth with catabolite repression medium (SOC) (10 mM NaCl, 2.5 mM KCl, 10 mM MgCl_2_, 10 mM MgSO_4_, 20 g L^−1^ tryptone, 5 g L^−1^ yeast extract + 2% glucose). Cells were incubated with shaking for 3 h at 37°C. One hundred microliters of culture was plated on LB10 plates supplemented with 200 μg mL^−1^ gentamicin to select for GFP and mCherry transformants. Bacterial stocks were derived from a single transformed colony.

### Competition assays.

To determine if prior exposure of P. aeruginosa to amoeba increased competitiveness when grown with amoeba, a competition assay was performed. with amoeba-adapted and nonadapted isolates derived on day 42 and were fluorescently tagged as described above. A. castellanii at a concentration of 1 × 10^5^ cells mL^−1^ was added to 24-well plates (Falcon) in 450 μL 1× M9 salts plus 0.01% glucose solution. Overnight cultures of GFP- or mCherry-labeled P. aeruginosa were grown in LB10 broth supplemented with 200 μg gentamicin at 37°C with agitation at 200 rpm. The amoeba-adapted::GFP and nonadapted::mCherry isolates or the amoeba-adapted::mCherry and the nonadapted::GFP isolates were mixed in equal proportions and added to the wells containing amoebae to a final bacterial cell concentration of 2 × 10^6^ cells mL^−1^. Each experiment was conducted in triplicate. The plates were incubated at room temperature with agitation at 60 rpm for 48 h before imaging on a Zeiss Z1 inverted wide-field microscope. Acquired images were deconvoluted in Autoquant X3 (Bitplane) before quantification of the relative red and green fluorescence in the field of view using Imaris 8 (Bitplane).

### Uptake and intracellular survival of P. aeruginosa in macrophages.

To investigate the dynamics of uptake and intracellular survival of day 42 adapted and nonadapted isolates within macrophages, overnight LB cultures of adapted and nonadapted P. aeruginosa isolates were added to RAW 264.7 macrophages (5 × 10^4^ cells/well in 96-well tissue culture plates) in DMEM without FBS at a multiplicity of infection (MOI) of 100:1. The infected cells were incubated at 37°C with 5% CO_2_. After coincubation for 1 h, the medium was removed and replaced with medium containing 100 μg mL^−1^ gentamicin to kill extracellular bacteria. Macrophages were washed with PBS and lysed at 3, 5, 7, 12, and 18 h postinfection, and CFU counts were performed to enumerate surviving intracellular cells. Propidium iodide (Thermo Fisher LIVE/DEAD cell viability kit) staining was done to determine the state of the host cells 24 h postinfection.

### Uptake and intracellular survival of P. aeruginosa in the presence of neutrophils.

P. aeruginosa (4 adapted and 3 nonadapted) isolates from overnight culture were washed once in PBS, diluted in PBS (OD = 0.1, ∼1 × 10^8^), and resuspended in complete medium (RPMI with 2% heat-inactivated autologous plasma) to experimental concentrations just prior to infection. Neutrophils were isolated from whole blood collected from healthy donors in lithium heparin Vacutainer tubes and separated using Polymorphprep (Axis-Shield) and centrifugation. Red blood cells (RBCs) were hypotonically lysed, and neutrophils were washed in Hanks balanced salt solution (HBSS) (without Ca^+^ or Mg^+^). Neutrophils were counted and resuspended at their final concentration in complete medium. In a 96-well plate, neutrophils were added to wells for challenge (neutrophil positive), and complete medium was added to control wells (neutrophil negative). P. aeruginosa was added to both polymorphonuclear leukocyte-positive (PMN^+^) and PMN^−^ wells at an MOI of 100:1 and incubated for 1 h at 37°C, 5% CO_2_. After coincubation for 1 h, bacterial survival was determined by serial dilution and plating on LB for enumeration. Uptake was determined by medium removal and replacement with medium containing 100 μg mL^−1^ gentamicin to kill extracellular bacteria. At the experiment endpoint, a sample of infection was taken and lysed in a new 96-well plate, followed by serial dilution and plating onto LB agar. CFU were determined by counting, and percent inoculum was determined as follows: (CFU of neutrophil-positive wells/CFU of neutrophil-negative wells) × 100. Counts were performed in triplicate, and results are the pooled means and standard errors of the means (SEM) from individual experiments using 3 different donors.

### Uptake and intracellular survival of P. aeruginosa in amoebae.

To investigate uptake and intracellular survival in amoeba, day 42 adapted and CF isolates were used to infect A. castellanii at MOI of 100:1. Briefly, amoebae (10^5^) were seeded onto 24-well plates in M9 medium, and bacterial cells (10^7^) were used to infect the amoebae. Following coincubation, the medium was removed and replaced with medium containing 100 μg mL^−1^ gentamicin to kill extracellular bacteria. Amoebae were washed with M9 and lysed at 2 and 4 h postinfection, and CFU counts were performed to enumerate surviving intracellular cells.

### Nematode survival assay.

To determine if P. aeruginosa adaptation to A. castellanii altered bacterial virulence, we tested the survival of C. elegans sp. Bristol N2 after feeding on P. aeruginosa. Axenic C. elegans organisms were obtained via the egg-bleach synchronization method, plated on NGM agar, and fed with heat-killed Escherichia coli OP50. L4-stage worms were resuspended in 1× M9 salts solution, and 10 to 30 worms were drop plated onto 35-mm dishes containing 2 mL fast- or slow-kill agar (53) containing lawns of pre-established amoeba-adapted or nonadapted isolates. Plates were incubated at 22°C, and worm numbers were scored by microscopy at 0, 4, 8, 24, and 48 h for fast-kill assays and once per day for slow-kill assays. Nematode toxicity was tested using 9 randomly selected P. aeruginosa isolates from each treatment and from 3 and 42 days. Nematode survival assays were repeated twice independently, and each experiment was performed in triplicate.

### Sequencing of P. aeruginosa populations and isolates and computational tools.

Amoeba-adapted and nonadapted P. aeruginosa populations and single isolates derived from these populations were sequenced to determine genotypic changes that occurred in response to coadaptation with amoebae. Genomic DNA was extracted from the parental wild-type strain and three replicates of amoeba-adapted and nonadapted populations derived from days 3 and 42. In addition, a total of 11 adapted and 3 nonadapted isolates derived from adapted and nonadapted populations from day 42 were also sequenced. Nine and three of these adapted and nonadapted isolates, respectively, from day 42 were same, and they were also used in all the phenotypic analyses. Genomic DNA was extracted using the QIAamp DNA minikit (Qiagen, Venlo, Netherlands) according to the manufacturer’s instructions. Sequencing libraries were prepared using the TruSeq DNA sample preparation kit (Illumina, San Diego, CA, USA), and sequenced on a MiSeq system (Illumina, USA). Reads were aligned to the P. aeruginosa PAO1 reference genome, and genetic variants, including nsSNPs, sSNPs, indels, and intergenic mutations, were detected using the breseq pipeline with a polymorphism frequency cutoff of 0.1 (54). The genetic variants were manually curated to filter out the mutations that were also present in the parental strain.

### Human ethics.

Ethical approval for whole-blood collection was obtained from the University of Wollongong Human Research Ethics Committee (HREC no. 08/250).

### Statistical analysis.

Phenotypic differences between amoeba-adapted and nonadapted isolates at specific time points (3, 24, and 42 days) were determined by analysis of variance (ANOVA), with amoeba adaptation (with or without A. castellanii) as a fixed factor and adaptation time (3, 24, or 42 days) as a random factor. Multiple testing was conducted using the Tukey *post hoc* test. All phenotypic data were log transformed [ln(*x* + 1)] prior to analysis to improve normality. *P* values of <0.05 were considered significant. Nematode survival curves were constructed with GraphPad Prism v 6.0 using the Kaplan-Meier method. Differences between nematode survival after exposure to amoeba-adapted and nonadapted P. aeruginosa isolates were determined using log-rank tests with significance given to *P* values of <0.05. Fisher’s exact test was performed to determine the statistical significance between shared and nonshared genes of adapted and nonadapted populations. Differences between neutrophil uptake and survival of amoeba-adapted and nonadapted strains were analyzed via Student’s *t* test.

### Data availability.

Sequence data related to this study are available in the NCBI Sequence Read Archive (SRA) under the BioProject accession number PRJNA753158.
