# Supplementary material for: Adaptation to an Amoeba Host Leads to Pseudomonas aeruginosa Isolates with Attenuated Virulence
Source: Appl Environ Microbiol. 2022 Mar 8;88(5):e02322-21. doi: 10.1128/aem.02322-21 (PMC8904051; doi:10.1128/aem.02322-21)
Supplement: Supplemental file 2 — Fig. S1 to S3. Download aem.02322-21-s0002.pdf, PDF file, 0.7 MB [file aem.02322-21-s0002.pdf]

Supplementary Information

Adaptation to an amoeba host leads to *Pseudomonas aeruginosa* isolates with attenuated virulence

Wai Leong[1¶], Wee Han Poh[1¶], Jonathan Williams[2, 3], Carla Lutz[1], M. Mozammel Hoque[4], Yan Hong Poh[1#a], Benny Yeo Ken Yee[1, 5], Cliff Chua[1, 5#b], Michael Givskov[1,6], Martina Sanderson-Smith[2,3] Scott A Rice[1,4,5*] and Diane McDougald[1,4*]

[1]Singapore Centre for Environmental Life Science Engineering, Nanyang Technological University, Singapore

[2]Illawarra Health and Medical Research Institute, Wollongong, Australia

[3]School of Chemistry and Molecular Biosciences, Molecular Horizons, University of Wollongong, Australia

[4]The ithree Institute, University of Technology Sydney, Sydney, Australia

[5]School of Biological Sciences, Nanyang Technological University, Singapore

[6]Costerton Biofilm Centre, Department of Immunology and Microbiology, University of Copenhagen, Copenhagen, Denmark

*Co-Corresponding authors

¶These authors contributed equally to this work.

[#a]Current address: Yale-NUS College, Singapore

[#b]Current address: Environmental Health Institute, National Environment Agency, Singapore

Email: RSCOTT@ntu.edu.sg and Diane.McDougald@uts.edu.au

Supplementary Figures

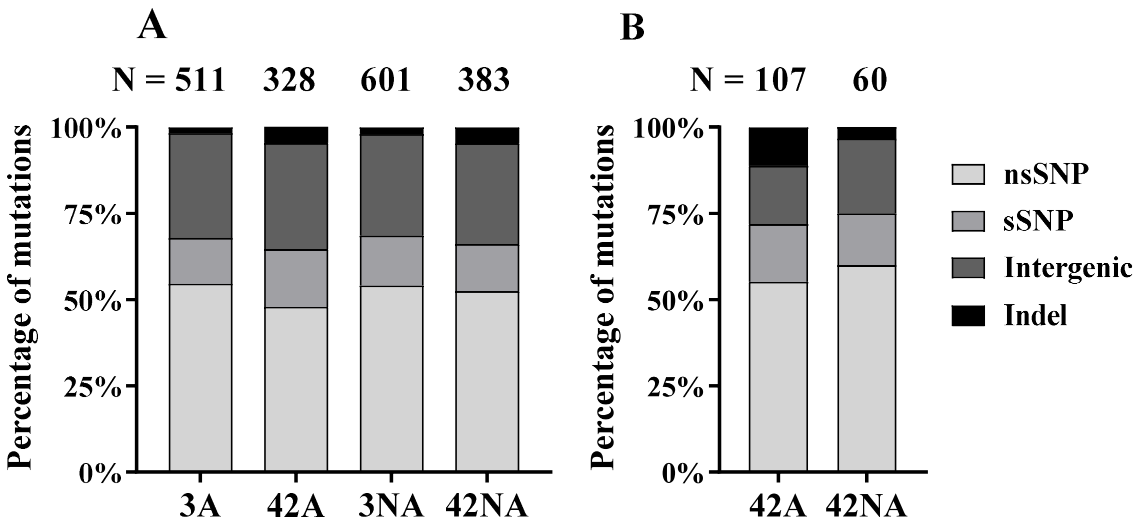

Supplementary Fig. 1. Number and types of mutations in adapted and non-adapted populations (A) and isolates (B) of *P. aeruginosa*. Shaded bars show the distribution of different types of mutations (nsSNPS, sSNPs indels and intergenic mutations) in the adapted (A) and non-adapted (NA) populations at day 3 and 42. The total number (N) of mutations are shown above each column.

Position	Mutation	Gene
188,489	intergenic (+41/-631)	*PA0164* → / → *PA0165*
557,675	I229I (ATC→ATA)	*PA0498* ←
1,167,377	intergenic (+107/-111)	*flgE* → / → *flgF*
1,172,959	coding (797/2052 nt)	*flgK* →
1,173,344	Y394* (TAC→TAG)	*flgK* →
1,189,846	coding (675-691/1209 nt)	*fleS* →
1,401,221	intergenic (-31/+191)	*PA1289* ← / ← *PA1290*
1,436,620	intergenic (+223/-43)	*PA1324* → / → *PA1325*
2,267,793	T524P (ACC→CCC)	*PA2069* ←
2,925,589	E67K (GAA→AAA)	*uvrC* ←
4,946,091	F17F (TTC→TTT)	*murG* ←
5,716,595	L87L (CTC→CTA)	*mdoH* ←
5,797,348	intergenic (+11/-17)	*PA5148* → / → *PA5149*
5,829,861	H89P (CAC→CCC)	*PA5177* →

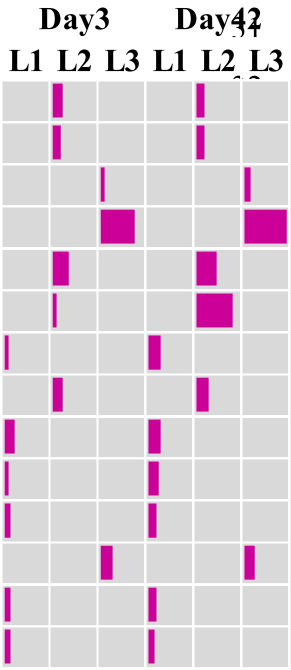

Supplementary Fig. 2. Persistent mutations in three replicates of amoeba adapted populations at day 3 and 42. Mutation position in genome, types of mutations and name of the affected gene are indicated in the first, second and third columns, respectively. Horizontal bars of the heatmap represent the frequency of respective mutations found in each replicate population (L1, L2, L3) with value ranges from 10 to 100%.

Position	Mutation	Gene
58,765	intergenic (-171/-21)	*PA0043* ← / → *exoT*
183,796	intergenic (+90/-26)	*PA0159* → / → *PA0160*
510,390	V177G (GTC→GGC)	*PA0451a* →
1,167,283	intergenic (+13/-205)	*flgE* → / → *flgF*
1,382,404	I201V (ATC→GTC)	*PA1271* →
1,436,615	intergenic (+218/-48)	*PA1324* → / → *PA1325*
1,724,377	F45S (TTC→TCC)	*sucA* →
2,038,540	N700N (AAT→AAC)	*PA1874* →
2,773,916	S1630F (TCC→TTC)	*PA2462* ←
2,814,767	*172* (TAG→TAA)	*PA2496* ←
3,096,900	D537N (GAC→AAC)	*PA2735* ←
3,268,790	L245P (CTC→CCC)	*PA2912* →
3,268,792	A246P (GCC→CCC)	*PA2912* →
3,977,180	intergenic (+69/-4)	*algJ* → / → *algF*
4,815,633	D197E (GAC→GAG)	*PA4292* →
4,843,609	intergenic (-57/-203)	*purU1* ← / → *mvaT*
4,967,848	E204G (GAG→GGG)	*PA4435* ←
5,486,084	H38H (CAT→CAC)	*PA4889* ←
5,616,596	N96I (AAC→ATC)	*waaL* →
5,882,197	T273P (ACC→CCC)	*pepP* ←
5,932,020	T220P (ACC→CCC)	*PA5266* ←

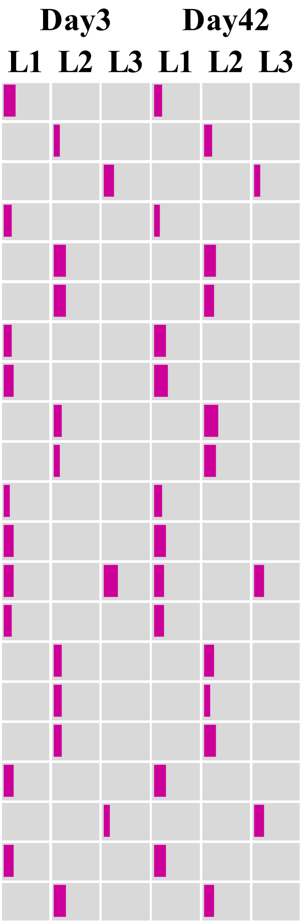

Supplementary Fig. 3. Persistent mutations in three replicates of non-adapted populations at day 3 and 42. Mutation position in genome, types of mutations and name of the affected gene are indicated in the first, second and third columns, respectively. Horizontal bars of the heatmap represent the frequency of respective mutations found in each replicate population (L1, L2, L3) with value ranges from 10 to 100%.
